# The alcohol harm paradox: is it valid for self-reported alcohol harms and does hazardous drinking pattern matter?

**DOI:** 10.1186/s12889-024-20530-9

**Published:** 2024-11-05

**Authors:** Ingeborg Rossow, Elin K. Bye

**Affiliations:** Dept of Alcohol, Tobacco and Drug Research, Norwegian Institute of Public Health, Oslo, Norway

**Keywords:** Alcohol Harm Paradox, Socio-economic position, Hazardous drinking pattern, Alcohol related harms

## Abstract

**Background:**

The alcohol harm paradox (APH) posits that alcohol harms are more prevalent in low socioeconomic position (SEP) groups compared to high SEP groups, when adjusted for alcohol consumption volume.

**Aim:**

We explored whether APH is valid for self-reported alcohol harms and whether SEP differences in hazardous drinking patterns may explain SEP differences in alcohol harms, accounting for consumption volume.

**Data and methods:**

We applied cross-sectional data from national population surveys in Norway, restricted to a subsample of past month drinkers aged 25–79 years (*n* = 8 171). Two binary outcome measures of self-reported alcohol-related harms were constructed from the AUDIT-10 items 4 through 10; alcohol dependence symptoms and alcohol related consequences. We separated two SEP groups based on education level: low versus high. Employing logistic regression models, we examined the extent to which SEP differences in alcohol-related harms were attributable to a more hazardous drinking pattern in terms of: (i) heavy episodic drinking and (ii) proportion of consumption volume by spirits, adjusting for consumption volume and demographic characteristics.

**Results:**

In the low SEP group, alcohol-related harms were significantly more prevalent as compared to the high SEP group when adjusted for gender and age, and more so after adjustment for consumption volume. Measures of hazardous drinking pattern and having a spouse could only to a minor extent account for the elevated risk of alcohol-related harm in the low SEP group. The findings were robust across various sensitivity analyses.

**Conclusion:**

Low SEP was associated with alcohol-related harm and a more hazardous drinking pattern but also with lower consumption volume. The findings support the validity of the alcohol harm paradox for self-reported alcohol harms.

## Introduction

Alcohol consumption is a major risk factor for the global burden of mortality and disease [1, 2]. Close to three million premature deaths per year are attributable to alcohol consumption [3]. Alcohol use is a risk factor for over 200 diseases or injuries, including infectious diseases, cancers, cardiovascular diseases, digestive diseases, traffic injuries and interpersonal violence [2]. Most of these diseases or injuries have multiple risk factors and hence are only partly attributable to alcohol use [4].

The risk of alcohol related harm increases with increasing alcohol consumption [5]. Thus, one might easily jump to the conclusion that alcohol related harms occur more frequently in population groups with higher alcohol consumption. However, this assumption does not seem to apply across groups with different socioeconomic position (SEP). Specifically, while it is often found that people with high education or high income (i.e. high SEP groups) tend to drink just as much or more than those in low SEP groups, alcohol related harms are often found to occur more frequently among those in low SEP groups [6–8]. In other words, there seems to be a positive social gradient in consumption volume, whereas an opposite (i.e. negative) social gradient is found for alcohol related harms. This relationship is termed the alcohol harm paradox (AHP) [9–11]. The implication is that disadvantaged groups suffer more harm per litre of alcohol compared to their advantaged counterparts [12, 13]. A similar observation is made at the population level: alcohol consumption tends to be higher in high income countries, but vulnerability to harms from alcohol is far higher in low and middle income countries compared to high income countries [1, 14]. Over the past decade, there has been a growing interest in AHP and research studies exploring the validity of the APH or/and possible explanations for the AHP [12]. In this study, we examine both.

Notably, what counts as alcohol related harm in this context, varies in the empirical literature. Several studies applied the AHP term with regard to outcomes for which alcohol use is among several contributing factors [12, 15]. In those studies, a negative social gradient in health outcome may well be due to SEP group differences in other important risk factors (e.g. smoking or unhealthy diet) than alcohol use. In the present study, we therefore delineate the focus to outcomes that are fully attributable to alcohol use; an approach which better reflects a paradox when SEP differences in consumption and harm go in opposite directions.

By way of introduction, we will briefly review the literature in two regards; (i) empirical evidence of the validity of the AHP, and (ii) empirical attempts to explain the AHP. Most of the literature pertains to individual level studies, which is also the focus in our study. Based on the review by Boyd and colleagues [12] and additional literature searches, 14 individual level studies, all from high income countries, reported findings relevant to the validity of AHP (i.e. they examined possible SEP differences in alcohol consumption/drinking behaviour and in alcohol related harm and whether the association between drinking and harm was moderated by SEP). Nine studies [16–24]; three from the UK, three from Finland, two from Sweden and one from Denmark, examined alcohol harm in terms of alcohol-attributable hospital admissions or/and mortality, and all these studies found empirical support for the AHP. Notably, these harms occur infrequently; in the magnitude of 2.4 per 1000 person years [24, 25]. Five studies [26–30] (from the UK, New Zealand, Denmark, Sweden and the Netherlands) examined self-reported alcohol related harms of various kinds; for example past year experience of injuries due to own drinking or symptoms of alcohol dependence. These are harms that are typically more frequently experienced in the general population (i.e. in the magnitude of 20–100 per 1000 person years [28]. Only two [26, 28] of the five studies examining self-reported harm found support for the AHP, suggesting that evidence for AHP is not entirely consistent and perhaps less so for self-reported harms that are more frequently experienced.

How can we explain the alcohol harm paradox? While scholars proposed several explanations or possible explanatory factors [12, 31], we will in this study focus on the possible roles of (i) a hazardous drinking pattern and (ii) having a spouse/partner. It is well established that a drinking pattern characterized by heavy episodic drinking (HED) adds to the risk of both acute and chronic alcohol-attributable harms at all consumption levels [32]. Thus, if HED occurs more frequently in low, as compared to high SEP groups, this could explain some of the elevated harm risk in low SEP groups. To this end, there is mixed empirical evidence for this assumption. Some studies found that very little or none of the SEP difference in alcohol-related harms was attributable to SEP difference in HED [17, 18, 20, 28], whereas other studies found that SEP difference in HED could explain much of the elevated alcohol harm risk in low SEP [22, 23]. Another aspect of hazardous drinking pattern pertains to beverage preference. There is some evidence that, given equal amounts being drunk, spirits consumption may be somewhat more closely associated with acute alcohol harms, as compared to wine or beer consumption [33–35]. Most important in this regard is probably the role of beverage type in various drinking situations; for instance that spirits seem to account for a larger fraction of the alcohol consumed on very heavy drinking occasions [33, 35]. The distribution of beverage type might therefore add to the assessment of a hazardous drinking pattern. Thus, if spirits are more often the beverage of choice in low SEP groups and accounts for a larger fraction of consumption, this aspect of drinking pattern might also contribute to explaining the negative social gradient in alcohol-related harms and the AHP. To this end, few studies examined this issue [12, 15]. A study by Gartner and colleagues [16] found that beverage type contributed very little to explain the SEP difference in alcohol-related harm.

Having a spouse or partner may possibly also moderate the consumption – harm association and in part explain the AHP. It is suggested that a spouse or partner may dampen the risk of alcohol-related harms by exercising informal social control of excessive drinking and providing social support [12, 31]. Thus, if those in high – as compared to low – SEP groups are more likely have a spouse or partner and thereby are more exposed to informal social control of their ways of drinking, this could also contribute to explaining the AHP. Among the few studies that examined this issue empirically, Mäkelä and Paljärvi [18] found that being married reduced the negative social gradient in alcohol-related harm to some extent.

In this study, we will add to this literature by examining the validity of the alcohol harm paradox for alcohol-attributable harms and by empirically exploring some possible explanations drinking patterns for the alcohol harm paradox. Specifically, we will examine whether self-reported alcohol related harms are more frequently reported by people in low as compared to high SEP groups when adjusted for alcohol consumption volume, and if so, we will examine whether hazardous drinking patterns and marital status may contribute to explaining socio-economic differences in alcohol-related harms when consumption volume is accounted for.

## Data and methods

### Sample

We employed data from seven annual population surveys on alcohol consumption, conducted in 2015 through 2021. Over this period, alcohol consumption level and prevalence of HED were stable in the Norwegian adult population [36]. The samples were drawn from the general Norwegian adult population (i.e. aged 16 through 79 years). Sampling procedures, measurements, and data collection methods (telephone interviews) were identical over time, and Statistics Norway conducted the surveys on behalf of the Norwegian Institute of Public Health. The total net sample comprised 15 211 respondents and the average response rate across the surveys was 59%. For this study, we used a subsample of respondents who had consumed alcohol in the past month (*n* = 9 540). We further restricted the sample to include those aged 25 years or older (*n* = 8 171) to identify those who have mostly completed their education (defining our SEP measure). The surveys were conducted according to the *Personal Data Act* and the *Statistics Act*. More details on sampling, data collection, and data curation are found elsewhere [37].

## Measures

The surveys included all AUDIT-10 questions [38], and in line with the study by Beard and colleagues [26] we constructed the following two outcome measures of alcohol-related harms from items 4 through 10: (i) dependence symptoms (score 2 + on items 4 through 6) and (ii) alcohol-related consequences (score 4 + on items 7 through 10) [39].

Socioeconomic position (SEP) was operationalized by employing a measure of highest completed education level with three categories: - mandatory education only (9 or 10 years in school, depending on age cohort), intermediate level (12 or 13 years in school), and university/university college level (at least 15 or 16 years in school/university). Other indicators of SEP, including household income and occupational status, were measured but not employed in the present analyses, mainly due to missing data. Moreover, an additional argument is that income and occupational status may well be affected by heavy drinking and severe alcohol problems over time (referred to as ‘downward social drift’) [40]. For example, low income or unemployment may be consequences of heavy drinking or alcohol problems. Thus, by examining whether people with these SEP characteristics suffer more harms from alcohol than others, we may introduce a circular argument. In comparison, we assumed education level is likely less affected by a heavy drinking career and therefore a preferable measure in assessment of AHP.

Alcohol consumption volume was calculated from data on beverage specific (i.e. beer, wine, spirits and alcopops/cider) consumption past 4 weeks. For each beverage, frequency by usual quantity (in number of drinks) was calculated into litres of pure alcohol.

We applied three indicators of hazardous drinking pattern. Two of these reflected HED and were measured as (i) Proportion of drinking occasions with 6 + units of alcohol^1^, and (ii) Proportion of drinking occasions being intoxicated^2^. A third indicator of hazardous drinking pattern was the proportion of total consumption by spirits. This was calculated from data on beverage specific consumption past 4 weeks. It should be noted that by using proportion measures (e.g. the proportion of drinking occasions having 6 + units or the proportion of consumption volume by spirits) these measures add to the volume of consumption and are less inter-correlated.

Having a spouse or partner was assessed by asking: “Are you married or cohabitant?”. Response categories were “Yes, married”, “Yes, cohabitant” and “No” and we used a dichotomous variable separating those who were married or cohabitant from the others. Demographic variables were age (continuous) and gender (binary).

## Statistical analyses

First, we examined whether there was support for the AHP by exploring SEP difference in harm outcome measures in bi-variate analyses, and we examined whether SEP groups differed by other demographic variables (age, gender, being married/cohabitant), and whether harm outcomes differed by demographics and drinking variables. Next, we employed the analytic approach used in previous studies of the AHP (e.g [16–18, 26, 28]). Specifically, we examined the SEP difference in alcohol related harm in logistic regression models. For each of the two outcome measures, we estimated models sequentially. In the first model, we regressed alcohol harm outcome on SEP, adjusting only for age and gender. Next, we included alcohol consumption volume in the model. In the further sequences, we explored whether hazardous drinking pattern (proportion of drinking occasions that were heavy episodic drinking and the proportion of consumption by spirits) could explain some of the SEP difference in alcohol-related harm. These variables were included by likelihood ratio criteria. Finally, we explored whether being married/cohabitant modified the SEP difference in alcohol related harm, again by including this variable by likelihood ratio criteria. For clarity, and in line with some previous studies [6], we contrasted the lowest (mandatory education only) and highest (university education level) SEP categories only (*n* = 4 828). Explanatory variables correlated only to a modest extent (all values *r* ≤ .32) and additional variance inflation factor (VIF) analyses (all values ≤ 1.23, mean: 1.10) suggested no concern for multicollinearity [41].

The results are based on weighted data (age, gender, educational level and geographic region) and the analyses were conducted in SPSS version 28.

## Sensitivity analyses

We ran sensitivity analyses to check for robustness of the findings in five regards. First, we analysed alcohol harms employing somewhat different measures; (i) we applied slightly different cut-offs for dependence symptoms and problems (i.e. a score of 1 + for dependence symptoms and 5 + and 6 + for problems) and (ii) we applied a sum-score measure across all 7 items on dependence symptoms and problems in linear regression models. Second, we included those with intermediate education level in a dichotomous SEP variable: low vs. intermediate/high education level. Third, as low SEP may increase the likelihood of drinking at extreme levels, as suggested by Lewer and colleagues [10], we included alcohol consumption as a categorical variable allowing for possible threshold effect of very high alcohol consumption. Fourth, an alternative measure of HED was proportion of occasions with subjective intoxication, and this measure was included in the models in sensitivity analyses. Finally, we explored whether the findings differed by gender, by running the analyses for men and women separately.

## Results

Altogether 700 respondents (9%) were categorized with alcohol-related consequences or alcohol dependence symptoms, based on their scores on the seven AUDIT items on alcohol harms (Table 1). There was some over-lap between the two outcomes; among those with alcohol-related consequences, 30% (148/495) also had dependence symptoms, and among those with dependence symptoms, 42% (148/353) also had alcohol-related consequences. For both outcomes, the proportion was highest in the low SEP group, and lowest in the high SEP group (Table 1). Alcohol consumption volume increased with increasing SEP, whereas the proportion of drinking occasions with subjective intoxication or 6 + units and the proportion of consumption volume from spirits were highest in the low SEP group and decreased with increasing SEP (Table 1). Demographic variables also varied by SEP; there was a lower proportion of women, a higher age and a lower proportion having a spouse in the low SEP as compared to the high SEP group (Table 1).

**Table 1:** Distributions of demographic characteristics, drinking behaviour and prevalence of alcohol harms for total sample and by three education level categories

Demographics, drinking behaviour and alcohol harms	Total sample (n = 8171)	Low education level (n = 1337)	Inter-mediate education level (n = 3344)	University education level (n = 3491)	Statistical test, p-value
**Alcohol harms**					
Alcohol-related consequences (4 + score), %	6.1	9.7	5.4	5.3	P < .001
Alcohol dependence symptoms (2 + score), %	4.3	5.4	4.5	3.8	P = .036
**Drinking behaviour**					
Alcohol volume, mean	0.32	0.29	0.32	0.34	P < .001
Prop. drinking occasions intoxicated, mean	25.7	32.4	29.8	19.3	P = .012
Prop. drinking occasions 6+, mean	16.9	19.4	17.6	15.2	P < .001
Prop. volume by spirits, mean	9.3	11.5	10.4	7.4	P < .001
**Demographics**					
Women, %	46.0	41.4	40.8	52.9	P < .001
Age, mean	49.1	48.9	51.9	46.6	P < .001
Married/cohabitant, %	75.2	69.8	75.3	77.2	P < .001

Note: Statistical tests for bi-variate associations between variables in 1st column and two categories of education level: lowest level vs. university level

Both outcome measures were significantly associated with drinking behaviour variables and demographics. Those with dependence symptoms or alcohol-related consequences had a higher consumption volume, a higher proportion of drinking occasions with subjective intoxication or 6 + units, and a higher proportion of consumption volume by spirits, compared to others (Table 2). Those with dependence symptoms or alcohol-related consequences had a lower proportion of women, a lower mean age, and a lower proportion living with a spouse, compared to others (Table 2).

**Table 2:** Drinking behaviour and demographic characteristics by presence of alcohol harms (*n* = 8 171)

Demographics, drinking behaviour and alcohol harms	Alcohol-related consequences (4+)		Alcohol dependence symptoms (2+)	
	No	Yes	No	Yes
**Drinking behaviour**				
Alcohol volume, mean	0.31	0.58	0.31	0.67
Prop. drinking occasions intoxicated, mean	24.1	51.2	24.3	56.2
Prop. drinking occasions 6+, mean	15.3	40.9	15.7	42.6
Prop. volume by spirits, mean	9.0	13.9	9.2	12.8
**Demographics**				
Women, %	47.0	30.6	46.9	27.5
Age, mean	49.7	40.1	49.5	41.4
Married/cohabitant, %	76.1	60.8	75.9	59.2

Note: all associations between alcohol harms on the one hand and drinking behaviour or demographic characteristics on the other were statistically significant (*p* < .001)

Next, we examined whether the SEP difference in the outcome measures were mediated by drinking patterns after adjustment for consumption volume. Overall, the results were quite similar for the two outcomes. There was a significant SEP difference (i.e. higher risk of alcohol harms) in the low SEP group, after adjusting for age and gender (Tables 3 and 4, Model 1). This difference was larger when adjusted for consumption volume (Tables 3 and 4, Model 2) and thereafter attenuated when adjusting for drinking pattern (i.e. proportion of drinking occasions with 6 + units and proportion of volume by spirits) (Tables 3 and 4, Models 3 and 4) and having a spouse (Tables 3 and 4, Model 5).

**Table 3:** Alcohol-related consequences^1^ regressed on education level (lowest vs. highest). Logistic regression models^2^ (*n* = 4 828)

	Model 1	Model 2	Model 3	Model 4	Model 5
**Low education level (95% CI)**	**1.94** **(1.52, 2.46)**	**2.23** **(1.74, 2.86)**	**2.06** **(1.60, 2.65)**	**2.01** **(1.56, 2.59)**	**1.94** **(1.51, 2.51)**
Alcohol volume		5.12	4.02	4.04	3.92
Proportion 6+			1.01	1.01	1.01
Proportion spirits				1.01	1.01
Married/cohabitant					0.68

Notes:

^1.^ Alcohol-related consequences: AUDIT items 7–10, score 4+

^2.^ All models were adjusted for gender and age

**Table 4:** Alcohol dependence symptoms^1^ regressed on education level (lowest vs. highest). Logistic regression models^2^ (*n* = 4 828)

	Model 1	Model 2	Model 3	Model 4	Model 5
**Low education level (95% CI)**	**1.37** **(1.02, 1.86)**	**1.58** **(1.15, 2.15)**	**1.43** **(1.04, 1.97)**	**1.40** **(1.02, 1.92)**	**1.35** **(0.98, 1.86)**
Alcohol volume		6.14	4.89	4.91	4.77
Proportion 6+			1.01	1.01	1.01
Proportion spirits				1.01	1.01
Married/cohabitant					0.71

Notes:

^1.^ Alcohol dependence symptoms: AUDIT items 4–6, score 2+

^2.^ All models were adjusted for gender and age

Sensitivity analyses showed that our findings were quite robust (data not shown). Applying outcome measures with slightly different cut-off values or applying a sum-score measure of alcohol related harms across all seven AUDIT items, did not change the overall findings substantially. Correspondingly, inclusion of those with intermediate education level in the SEP variable did not alter the overall findings, however, the magnitude of the SEP difference was somewhat less in these analyses. Bivariate analyses showed that the proportion with alcohol harm was particularly elevated in the upper consumption category (above the 90th percentile). However, the proportion of drinkers in this upper category did not differ by SEP and inclusion of the categorical consumption volume measure did not alter the findings. Moreover, inclusion of the variable on proportion of drinking occasions with HED in terms of subjective intoxication (rather than 6 + units) did not improve model fit significantly, but otherwise the overall findings were much the same. Finally, gender specific analyses did also show the same pattern of results, as reported from the main analyses.

## Discussion

In the adult population in Norway, we found that self-reported alcohol-related harms were significantly more prevalent among those in low, as compared to high, SEP. The difference was more prominent when considering the higher volume of consumption with higher SEP. A hazardous drinking pattern, in terms of higher proportion of heavy episodic drinking occasions and higher proportion of spirits consumption attenuated this difference to some extent, as did having a spouse.

Our findings add to a small literature with mixed findings regarding self-reported alcohol-related harms [12]. Several previous studies demonstrated a negative social gradient in severe alcohol-related harms (i.e. hospital admissions or mortality) as well as a positive social gradient or no SEP difference in alcohol consumption volume [16–24], in line with the AHP, and our study – examining less severe harms – adds further empirical support to the alcohol harm paradox [12]. In line with several previous studies [22, 23, 29], we found that a hazardous drinking pattern with regard to heavy episodic drinking was more prominent in low as compared to high SEP and could thereby explain some of the SEP difference in alcohol harms. Stronger preference for spirits did not contribute much to explain the SEP difference in alcohol-related harm in our study, which is in line with a previous study finding [16]. Finally, in line with the study by Mäkelä and colleague [18], we found that having a spouse contributed to attenuate the SEP difference in alcohol-related harms. Most importantly, in most models, a substantial SEP difference in alcohol harms remained after adjustment for several suggested explanatory factors, a finding that mirrors those from previous studies of the alcohol harm paradox [12, 31].

Thus, it is still a puzzle why alcohol harms occur more frequently in low SEP groups and hence why these groups seem to be so more vulnerable to the effects of drinking than those in high SEP groups. A possible explanation is that important confounders were not accounted for, in our study and in previous studies. One suggested confounder is access to treatment services. If people in low SEP groups have less access to health care, they may be more likely to develop alcohol problems than those in high SEP groups [12]. While it may be argued that the AHP seems to be valid also in Nordic countries [18, 22, 23, 29], where the social welfare systems (are supposed to) compensate for social inequities in access to care and services, a positive social gradient in use of primary health care among people with health problems is found also here, for instance in Norway [42]. Differential use of health services may therefore confound the observed elevated risk of alcohol harm in the low SEP group. Another possible unmeasured confounder is long-term alcohol exposure. This study employed cross-sectional data on recent drinking behaviour on the one hand and alcohol harms associated with long term exposure on the other. Thus, if current drinking behaviour reflects accumulated alcohol exposure differently across SEP groups, this may have bearing on the alcohol harm paradox by confounding the SEP – harm association. A recent longitudinal study from Sweden lends support to this assumption, finding that stability of drinking careers differed by baseline SEP [43].

Several study limitations should be noted. First, heavy drinkers are likely under-represented in the general population sample [44], which implies downward biased rates of alcohol harm. If such under-representation varies by SEP, it will distort the SEP-harm association. Second, it is possible that the validity of self-reported drinking behaviour and self-reported harms differ by SEP. There are two reasons for this. The way concepts are understood or interpreted in a survey may possibly differ by SEP, and systematic under-reporting reflecting a social desirability bias, may also differ by SEP. If so, the observed SEP-harm associations may be biased, one way or the other. Regarding possible SEP-difference in under-reporting, Peña and colleagues [20] measured alcohol consumption by self-reports and bio-markers and concluded that differential bias in self-reported consumption is not a likely explanation for the AHP. Third, several possible confounders, as discussed above, were not assessed in this study, which may contribute to explain the observed AHP. Fourth, we examined only individual SEP characteristics, and it is possible that inclusion of contextual factors (e.g. disadvantaged neighbourhood) to the analysis might add to our understanding of the AHP complexity. Moreover, we only employed one SEP marker (educational attainment), and hence we could not assess robustness of findings across various SEP markers. Nor could we assess whether income or occupational status was a stronger effect modifier compared to education level, which was a finding reported in a previous study [17] and in line with the ’downward social drift’ assumption. Fifth, alcohol harms were limited to those identified by the AUDIT-10 instrument, which mainly pertains to harms to the drinker. Finally, the study sample was relatively small for examining SEP-difference in low-prevalent outcomes with sufficient precision to compare the relative importance of possible explanatory factors. It is also likely that the non-significant adjusted association between SEP and dependence (Table 4, Model 5) reflects insufficient statistical power.

We suggest some implications from this study. First, further studies are needed to fill some of the gaps in our understanding of social inequality in alcohol consumption and related harms. These studies could include, for instance: - longitudinal design assessing long term alcohol exposure and harm risk over time, - a broader range of alcohol harms including social harms and harms to others, - population samples from low - or middle-income countries.

Second, alcohol consumption contributes not only significantly to the overall health burden [1, 2] but also to social inequality in health [45]. Hence, our results, along with other empirical evidence in support of the AHP, suggest a potential for reducing some of the social health inequality by measures that effectively reduce alcohol consumption and related harms. There is good evidence that alcohol control policy measures restricting affordability and physical availability of alcohol are effective to curb consumption and harm at the population level [46] and some studies have suggested a *potential* for price policy to reduce social inequality in hazardous drinking and/or related harms (e.g [47–49]. Interventions that specifically select and target low SEP groups could be stigmatising, and hence universal interventions with evidence of stronger impact in disadvantaged groups, would be a preferable strategy. In particular, some findings from natural experiment and modelling studies suggest minimum unit pricing of alcohol may be more effective at reducing alcohol harms among people in low SEP groups [50], although the evidence is not consistent [51].

Third, in a global perspective, the elevated vulnerability to harms from alcohol in low- and middle-income populations [1], calls for particular concern. Many of these populations have become important markets for transnational alcohol corporations and it is therefore an urgent need for international collaboration and strengthening of effective alcohol policies globally [46].

## Conclusion

Alcohol-related harms were more prevalent in low SEP groups after adjusting for alcohol consumption volume. The findings support the validity of the alcohol harm paradox for self-reported alcohol harms. Aspects of drinking pattern and having a spouse could only to some extent explain the SEP difference in alcohol harms. However, some potential confounders were not accounted for.

## Data Availability

The datasets analysed in this study can be obtained from the Norwegian Agency for Shared Services in Education and Research : Find research data (sikt.no).

## Footnotes

1 This was calculated from a series of questions on graded frequency consumption past 4 weeks. For each of the following amounts of alcohol, the respondents were asked how many times they had drunk this amount in the past 4 weeks: 1–2 drinks, 3–5 drinks, 6–11 drinks, and 12 or more drinks. The six frequency categories ranged from ‘About every day’ to ‘Once in the past four weeks’, which were given values between 28 and 1 to obtain a semi-continuous measure of number of drinking occasions.

2 This was calculated from two semi-continuous measures: frequency of drinking in the past 12 months and frequency of drinking to subjective intoxication in the past 12 months.
